# Polygenic and single-locus selection on BMI during Polynesian expansion

**DOI:** 10.1038/s10038-025-01441-y

**Published:** 2025-12-15

**Authors:** Hanako Miwa, Mariko Isshiki, Izumi Naka, Ryosuke Kimura, Tsukasa Inaoka, Yasuhiro Matsumura, Jun Ohashi

**Affiliations:** 1 Graduate School of Science, The University of Tokyo, Bunkyo-ku, Tokyo Japan; 2 Department of Genetics, Albert Einstein College of Medicine, New York, NY USA; 3 Graduate School of Medicine, University of the Ryukyus, Ginowan-shi, Okinawa Japan; 4 Faculty of Agriculture, Saga University, Saga-shi, Saga Japan; 5 Faculty of Health and Nutrition, Bunkyo University, Chigasaki-shi, Kanagawa Japan

**Keywords:** Population genetics, Risk factors

## Abstract

The ‘thrifty’ variant hypothesis, which posits that certain genetic adaptations promoting efficient energy storage during periods of food scarcity, has been invoked to explain the high prevalence of obesity in modern human populations. Although several candidate variants have been proposed, the timing and effects of these variants on body mass index (BMI) in specific populations remain poorly understood. In this study, we performed whole-genome sequencing of 22 Tongan individuals. A previous study identified the rs373863828-A variant in the *CREBRF* gene as a target of positive selection in Samoans based on iHS analysis. Here, we replicated this signal in Tongans, confirming that this variant has been subject to adaptive pressures more broadly across Polynesian populations. Using the CLUES program, we inferred the allele frequency trajectory of rs373863828-A in Tongans, revealing a marked increase over the past ~100 generations that temporally aligns with the period of Polynesian maritime expansion. The trajectory of the Polygenic Score (PS) showed an increase in the PS for BMI in ancestors of Tongans between 150 and 50 generations ago, followed by a recent decline. Analysis of polarized trait integrated haplotype scores detected significant polygenic selection favoring lower BMI in recent generations. Our findings suggest that the *CREBRF* variant underwent strong positive selection during oceanic dispersal, while numerous modest-effect variants collectively contributed to adaptation to food-limited environments during long sea voyages. More recently, however, selection pressures may have shifted toward lower BMI, indicating a potential evolutionary mismatch between past adaptations and modern environments.

## Introduction

The ‘thrifty’ variant hypothesis has been proposed to explain the high prevalence of obesity in the island populations of Remote Oceania [1]. This hypothesis proposes that genetic variants associated with metabolic efficiency or obesity conferred a selective advantage under conditions of limited food availability, particularly during long-distance sea voyages [1, 2]. Such voyages occurred during the Pacific expansion, a major human dispersal event in the late Holocene, in which Austronesian (AN)-speaking populations—likely originating from Taiwan—began migrating through Island Southeast Asia into Oceania. These populations first reached the Bismarck Archipelago in Near Oceania ~3500 years ago, and from there continued eastward into the more isolated islands of Remote Oceania [3–14]. The most intense selective pressures related to food scarcity and maritime endurance were likely encountered during this latter phase of the Pacific expansion—from Near to Remote Oceania—when long ocean crossings and limited resources would have strongly favored genotypes promoting energy storage and metabolic efficiency.

Among the many genetic variants associated with obesity, rs373863828-A in the *CREBRF* gene stands out as the most compelling example consistent with the ‘thrifty’ variant hypothesis [15–18]. This variant is significantly associated with BMI in Polynesians, particularly in Samoans [17] and Tongans [18], and also shows a strong signal of positive natural selection in Samoans [17]. While numerous obesity-associated variants have been identified across different populations through genome-wide association studies (GWAS), rs373863828-A remains one of the very few for which robust evidence of natural selection has been detected [17, 19]. Thus, it represents perhaps the strongest known candidate for a ‘thrifty’ variant in Oceanic populations.

Beyond this single-variant example, metabolic and obesity-related traits, such as BMI and Type 2 diabetes, are widely recognized as polygenic, with numerous associated variants identified across populations [19–21]. Among them, variants in *ADRB2, LEPR* and *PPRAGC1A*, repeatedly linked to obesity in global studies, have also shown significant associations in Oceanic populations [22–24]. In GWAS conducted in Samoans, rs373863828-A is virtually the only variant that reaches genome-wide significance [17]. The absence of other significant signals is likely due to small sample sizes and limited statistical power. Therefore, the potential role of modest-effect variants in population-level adaptation should not be overlooked.

Indeed, recent studies have suggested that polygenic adaptation—where selection acts on many small-effect variants rather than a single large-effect allele—may have shaped metabolic traits such as lipid metabolism in Oceanic populations [25]. However, the timing of these adaptive processes and their temporal relationship to the human expansion into Remote Oceania remain poorly understood. Moreover, it is still unclear to what extent these adaptations were driven by newly acquired alleles during the dispersal or by standing genetic variation already present in ancestral populations.

In this study, we conducted whole-genome sequencing of 22 Tongan individuals. The main objective was to investigate the evolutionary history of genetic adaptations related to BMI in Tongans and determine when these adaptations arose during the dispersal of their ancestors into Remote Oceania. We examined signals of positive selection on variants reported to be associated with BMI. Additionally, we reconstructed the evolutionary history of polygenic scores (PS) for BMI to determine when genetic adaptations influencing BMI occurred. Furthermore, we applied a method comparing polarized integrated haplotype scores (iHS) to detect signatures of polygenic adaptation by identifying genomic regions where multiple BMI-associated variants exhibit coordinated patterns of recent positive selection, an approach that has been recently applied in studies of Oceanic populations [25].

## Materials and methods

### Sample collection and approvals

Blood samples were collected from 22 Tongan males after obtaining informed consent from each participant. This study was approved by the institutional review boards of the Graduate School of Medicine and the Graduate School of Science, The University of Tokyo.

### DNA extraction and sequencing

Genomic DNA was isolated from peripheral lymphocytes using a commercial kit according to the manufacturer’s instructions (QIAamp, Qiagen, Hilden, Germany). Library construction, sequencing, alignment and variant calling were performed by Macrogen Japan (Kyoto, Japan). Genomic library was constructed with the TruSeq DNA PCR-Free library prep kit (Illumina) according to the Illumina TruSeq Nano DNA library preparation guide or TruSeq DNA PCR-free library preparation guide to obtain a final library of 300–400 bp average insert size. Sequencing was conducted on the DNA libraries using the NovaSeq 6000 System (Illumina), according to the manufacturer’s protocol. The raw base call binary files (BCL/cBCL) were converted into FASTQ using illumina package bcl2fastq2-v2.20.0. The paired-end sequences generated are mapped to the human genome using Isaac aligner (iSAAC-04.18.11.09) [26].where the reference sequence is the UCSC assembly hg19. The variant calling was performed using Strelka version 2.9.10 (Illumina) [27]. The genotype data of the Tongan individuals are available from the authors upon reasonable request for research purposes only.

### Genetic structure analysis

The genome-wide dataset of 22 individuals from Tonga was processed and merged using VCFtools [28], BCFtools [29], and PLINK [30], and subsequently phased with Beagle [31]. To investigate population structure, this Tongan dataset was then combined with 2504 individuals from the 1000 Genomes Project phase 3 [32]. After merging these datasets, we obtained a genome-wide dataset comprising 2526 individuals from 27 populations and 6,921,230 autosomal biallelic SNPs. Subsequently, we conducted linkage disequilibrium (LD) pruning with the following parameters: window size of 50, step size of 10, and an ${r}^{2}$ threshold of 0.1 (–indep-pairwise 50 10 0.1). This pruning process reduced the number of SNP markers to 6,252,857. Principal component analysis (PCA) was performed on this pruned dataset using PLINK v1.9 [30] to identify potential population substructures. Additionally, ADMIXTURE analysis was performed using ADMIXTURE v1.3.0 [33] on a subset including East Asian populations (represented by CHB; *n* = 103), European populations (represented by CEU; *n* = 99), and Tongans (*n* = 22). Different values of *K* (from *K* = 1 through *K* = 5) were explored on the pruned SNP set to determine the optimal value of *K*. The optimal *K* value was identified as *K* = 3 by the cross-validation procedure implemented in the ADMIXTURE software [33]. Through this ancestry screening process using *K* = 3 components, we verified that no CEU ancestry components were detected among 22 Tonga individuals. The results were visualized using the ggplot2 package in R [34]. This merged dataset was used only for exploratory population-structure analysis and not for any selection-related analyses. All subsequent analyses, including relatedness filtering and selection analyses, were conducted exclusively using the original Tongan genotype data, which were independent of the 1000 Genomes dataset.

### Kinship analysis

Relatedness filtering was conducted using VCFtools v0.1.16 (–relatedness2) [28] with a kinship coefficient threshold of 0.0625, ensuring that all Tongan individuals were unrelated at or closer than the first-cousin level.

### Testing signals of positive selection on BMI- or obesity-associated variants

For this analysis, we selected four SNPs (*CREBRF* rs373863828, *ADRB2* rs34623097, *LEPR* rs1137101, and *PPARGC1A* rs8192678) that have previously been reported as BMI- or obesity-associated in Oceanian populations [15–18, 22, 24, 35]. The ancestral and derived allelic states of each SNP were determined using the Ensembl genome browser, and the BMI- or obesity-associated alleles were identified based on information from previous studies and the SNPedia database [36] (Supplementary Table S1).

To test for signatures of positive selection on these BMI- or obesity-related variants, we calculated extended haplotype homozygosity (EHH) and integrated haplotype score (iHS) using the software selscan [37]. The procedure used closely followed that of Minster et al. (2016). First, EHH values were calculated to assess how long regions of the genome remain identical around a selected allele [38]. When positive selection occurs, EHH values tend to remain high over a broader region. Then, iHS values were computed as the log ratio of the integrated EHH values for the derived and ancestral alleles [39] and were standardized across 25 allele frequency bins genome-wide. A large positive iHS suggests that the derived allele has risen in frequency due to selection. The selscan software was run with the ‘–keep-low-freq’ option, and other settings were left at default. EHH plots were created using the ggplot2 [34], ggsci [40], and reshape2 [41] packages in R.

### Estimation of allele frequency trajectories

Relate [42, 43] and CLUES [44] were used to infer the frequency trajectories of BMI- or obesity-related variants (Supplementary Table S1). First, genetic data from 22 Tongan individuals were processed using the Relate software package. Phased VCF files were converted using the RelateFileFormats utility in ConvertFromVcf mode and filtered using the PrepareInputFiles.sh script, with human ancestor sequences (GRCh37/hg19, Ensembl release e59) and pilot mask files (20140520). Genealogical trees were reconstructed using the Relate algorithm in ‘All’ mode, with a generation time of 28 years (the default setting in Relate). The analyses assumed a default mutation rate of 1.25 × 10^−8^ per base pair per generation and an effective population size of 10,000. The genetic recombination map from the combined b37 build was used to obtain the recombination landscape across chromosomes. The human ancestor sequences, pilot mask files, and genetic recombination maps were obtained from the Relate Documentation Website [42, 43, 45]. Genealogical trees were constructed separately for each autosome. Historical effective population size was estimated using the EstimatePopulationSize.sh script from Relate. The estimation was performed iteratively across all autosomes with 10 iterations to refine the coalescent model. For the loci of interest, branch lengths were sampled using the SampleBranchLengths.sh script. This process generated 100 samples of branch lengths at designated positions, using the previously estimated coalescent model. The sampling was limited to specific base pair positions of interest for targeted analysis of potentially selected regions. Then, CLUES framework was used to reconstruct allele frequency trajectories [44]. For each site of interest, trajectories were inferred using the sampled branch lengths and the coalescent model estimated by Relate via the inference.py script, which simultaneously estimated the selection coefficient. These trajectories were visualized using the plot_traj.py script. Finally, genealogical relationships at specific loci were visualized using the TreeView.sh script from Relate.

### Reconstruction of trajectory of polygenic scores

To reconstruct the historical trajectory of polygenic scores (PSs) for BMI, the results from Relate [42, 43] and CLUES [44] were applied within the framework proposed by Edge and Coop [46]. The population-average polygenic score $Z$ at time $t$ was estimated using the formula $Z(t)={\sum }_{i}{\beta }_{i}{p}_{i}(t)$, where ${\beta }_{i}$ represents the effect size of the focal allele at SNP $i$, and ${p}_{i}(t)$ denotes the allele frequency at time $t$. In this study, ${p}_{i}(t)$ was estimated by integrating the Relate and CLUES algorithms. The preparatory steps for allele frequency estimation followed the same procedures described above (see the subsection “Estimation of allele frequency trajectories”). Based on our observation that rs373863828-A exhibited a marked increase in allele frequency over approximately the past 100 generations, we focused on three 50-generation periods in this analysis to capture selection dynamics before, during, and after this period. Accordingly, when utilizing the inference.py script, we specified epochs of 0–50, 50–100, and 100–150 generations as periods during which selection was allowed using the ‘–timeBins’ option. The GWAS summary statistics for BMI were obtained from the East Asian data of the BMI Exome Array Summary Statistics (GIANT East Asian dataset) [47]. We used this dataset because no Polynesian or Oceanian GWAS with sufficient SNP coverage and sample size was available; ~1000 SNPs overlapped between our Tongan data and the GIANT East Asian dataset, compared with only about 400 with the BioBank Japan (BBJ) dataset [48].

To assess whether BMI-associated alleles identified in East Asian populations are applicable to Oceanians and have consistent directional effects, we performed a regression analysis examining whether PSs calculated based on the GIANT East Asian dataset explain variance in BMI among the Tongan individuals. The validity of the PS was evaluated by comparing three regression models using (i) age only, (ii) PS only, and (iii) both age and PS as predictors of BMI. To account for possible age-related effects on BMI prediction, these model comparisons were conducted across four age-based subsets: all individuals (*n* = 22), <55 years (*n* = 15), <50 years (*n* = 12), and <45 years (*n* = 10).

### Polygenic adaptation

To investigate polygenic adaptation on BMI and other traits primarily associated with obesity and metabolic function, the significance of differences in polarized trait-integrated haplotype scores (tiHSs) was tested against those of randomly selected SNPs matched for derived allele frequency (DAF), local recombination rate, and genomic evolutionary rate profiling (GERP) scores [25]. The tiHSs were calculated by polarizing the iHS statistics with the corresponding GWAS effect sizes. The iHS values for the derived alleles were calculated using selscan [37]. To calculate tiHS values, we included SNPs that were suggestively significant ($P < {5.0\,\times 10}^{-2}$) in GWAS and for which iHS statistics were available in the Tongan population. Each SNP was classified as either ‘trait-increasing’ or ‘trait-decreasing’ based on the sign of the effect size ($\beta$) of the focal allele, as reported in the GWAS summary statistics. During the calculation of tiHS, the derived allele in the iHS dataset was aligned with the effect allele in the GWAS summary statistics. In cases where the GWAS effect allele corresponded to the ancestral allele, the sign of the effect size ($\beta$) was inverted to ensure consistency between the two datasets. GWAS summary statistics for BMI were obtained from the GIANT consortium [47], and those for BMI and other metabolism-related traits and blood biochemical parameters were obtained from the BBJ dataset [48]. From the BBJ dataset, we focused on metabolism-related traits and blood biochemical parameters that retained at least 100 genome-wide significant SNPs in our Tongan dataset, to ensure the reliability of the tiHS estimates and to minimize potential biases due to limited SNP representation. As a result, the traits that met these criteria and were retained for analysis were BMI, HDL cholesterol, hematocrit, hemoglobin, LDL cholesterol, mean corpuscular hemoglobin, platelet count, red blood cell count, serum creatinine levels, type 2 diabetes, and white blood cell count (Table 1). It should be noted that the four BMI- or obesity-associated variants listed in Supplementary Table S1 (*CREBRF* rs373863828, *ADRB2* rs34623097, *LEPR* rs1137101, and *PPARGC1A* rs8192678) were not included in the tiHS analysis for any traits.

**Table 1:** Evaluation of polygenic adaptation on metabolic and hematological traits

GWAS Traits	Number of suggestively significant SNPs reported	Number of SNPs retained in our Tongan dataset	Direction of selection	*P*-value	*P*-value (adjusted)
BMI (GIANT East Asian dataset)	1467	1041	Decreasing	2.0 × 10^−7^	2.4 × 10^−6^
BMI (BBJ dataset)	601	383	Decreasing	1.4 × 10^−3^	4.0 × 10^−3^
HDL cholesterol	318	190	Decreasing	1.2 × 10^−2^	1.7 × 10^−2^
Hematocrit	374	241	Decreasing	7.4 × 10^−3^	1.1 × 10^−2^
Hemoglobin	746	424	Decreasing	3.2 × 10^−2^	3.8 × 10^−2^
LDL cholesterol	188	92	Decreasing	2.0 × 10^−1^	2.2 × 10^−1^
Mean corpuscular hemoglobin	652	398	Decreasing	1.7 × 10^−3^	4.0 × 10^−3^
Platelet count	627	391	Decreasing	6.7 × 10^−4^	2.7 × 10^−3^
Red blood cell count	501	312	Decreasing	1.8 × 10^−5^	1.1 × 10^−4^
Serum creatinine levels	492	304	Decreasing	4.3 × 10^−1^	4.3 × 10^−1^
Type 2 diabetes	307	307	Decreasing	2.6 × 10^−3^	5.3 × 10^−3^
White blood cell count	472	295	Decreasing	3.9 × 10^−3^	6.6 × 10^−3^

Results of trait-integrated haplotype score (tiHS) analysis for polygenic adaptation in Tongans. Mean tiHS values for SNPs associated with each trait were compared with an empirical null distribution generated from 10,000,000 random replicates to evaluate the direction and strength of selection. Two-sided empirical *P*-values were calculated and adjusted for multiple testing using the Benjamini–Hochberg (BH) method. Adjusted *P*-values < 0.05 were considered significant

*HDL* high-density lipoprotein, *LDL* low-density lipoprotein, *BMI* body mass index

To evaluate the direction and strength of selection on each trait, we first calculated the mean tiHS of all SNPs associated with the focal trait. We then generated an empirical null distribution by randomly sampling the same number of SNPs as in the trait-associated set, repeating this process 10,000,000 times. To assess whether selection acted to increase or decrease the trait, the observed mean tiHS was compared with the distribution of mean tiHS values obtained from 10,000,000 random replicates. If the observed mean tiHS was greater than the average of the random replicates, selection was inferred to have acted to increase the trait; conversely, if the observed mean was smaller, selection was inferred to have acted to decrease it. For two-sided testing, the absolute values of both the observed mean tiHS and the random sample means were used. The empirical two-sided *P*-value was calculated as *P* = (1 + *r*) / (*N* + 1), where *r* is the number of random replicates whose absolute mean tiHS values were greater than or equal to the absolute observed value, and *N* = 10,000,000 is the total number of replicates. *P*-values were calculated for the 12 traits listed in Table 1 and subsequently adjusted for multiple testing using the Benjamini–Hochberg (BH) method. Adjusted *P*-values less than 0.05 were considered statistically significant.

## Results

**Fig. 1:**
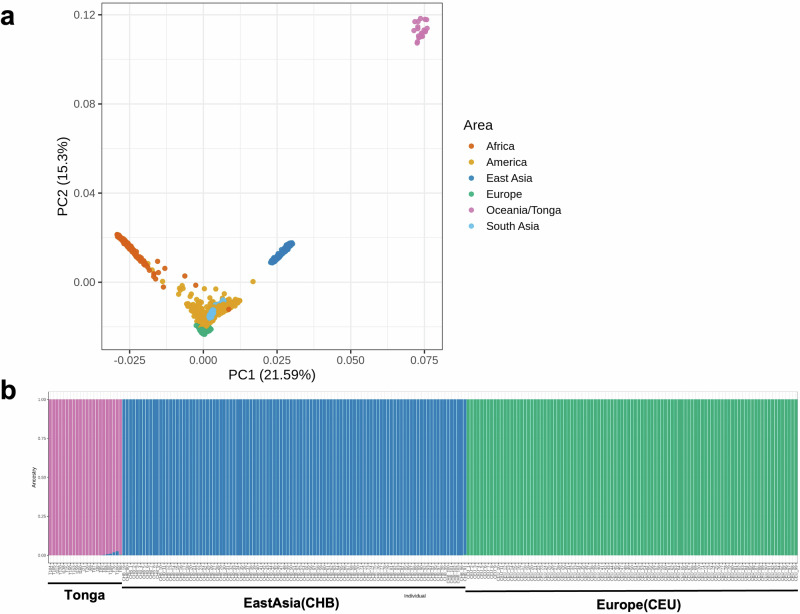
Population genetic analysis of Tongan individuals. **a** Principal component analysis (PCA) plot showing genetic relationships between Tongan individuals (pink) and reference populations from different continental regions(1000 Genome Project Phase3). Colors represent different geographic regions: Africa (red), America (yellow), East Asia (blue), Europe (green), Tonga (pink), and South Asia (light blue). **b** ADMIXTURE analysis showing population structure across individuals, with colors indicating different ancestral components (*K* = 3)

Relatedness analysis confirmed that all 22 Tongan individuals were unrelated males, and these individuals were subsequently used for the analyses. A PCA plot including the Tongans and populations from the 1000 Genomes Project dataset (phase 3) is shown in Fig. 1a. The Tongan individuals formed a distinct cluster in close proximity to East Asian populations. To investigate individual ancestry proportions, we performed ADMIXTURE analysis for K values ranging from 1 to 5, with *K* = 3 yielding the lowest cross-validation error. The results for *K* = 3 are shown in Fig. 1b. This analysis revealed clear population structure separation between East Asians (CHB), Europeans (CEU), and Tongans, indicating that the 22 Tongan individuals analyzed here form a genetically homogeneous group representing predominantly Polynesian ancestry, with minor East Asian-related components detected in a few individuals.

**Fig. 2:**
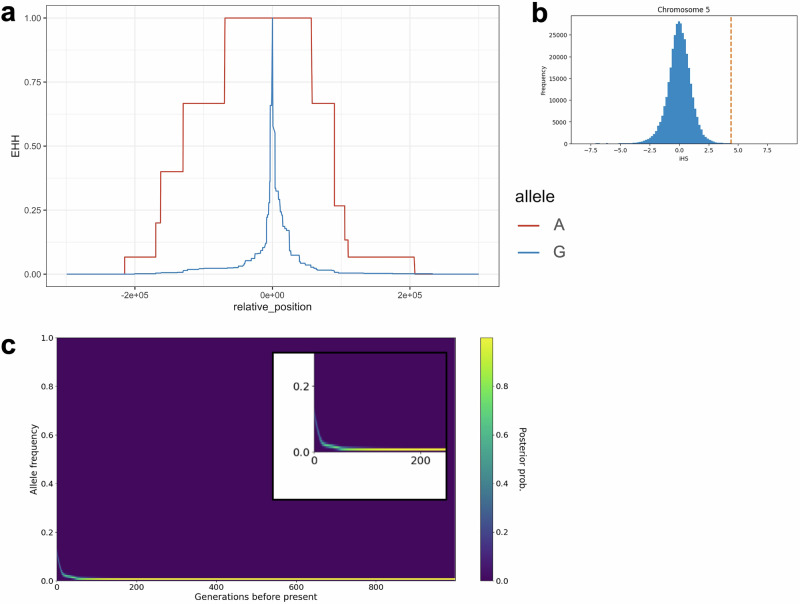
Detection and timing estimation of natural selection on rs373863828-A in Tongan population. **a** EHH decay plot for derived (A: red) and ancestral (G: blue) alleles of rs373863828 in the *CREBRF* gene. iHS = 4.40, ${P}=1.1\,\times {10}^{-5}$. **b** Distribution of iHS on chromosome 5. **c** Heatmap showing allele frequency changes over 1000 generations before present, indicating potential selection pressure on this variant

A missense variant, rs373863828-A in the *CREBRF* gene, has been reported as a ‘thrifty’ variant associated with increased BMI in Samoans [17]. Our previous study also confirmed the association between this variant and BMI in Tongans [18]. Given the critical importance of this variant as a leading candidate ‘thrifty’ allele supporting the metabolic adaptation hypothesis during Pacific colonization, we investigated whether the signatures of positive selection observed in Samoans [17] could be replicated in the Tongan population. To evaluate recent positive selection on this variant in Tongans, we measured EHH and iHS across chromosome 5. The iHS analysis revealed that the haplotype homozygosity of rs373863828-A was significantly more extended than that of the ancestral allele rs373863828-G (iHS = 4.40, *P* = 1.1 × 10⁻⁵) (Fig. 2a). To place this signal in a broader genomic context, we plotted the chromosome-wide distribution of iHS values for chromosome 5 (Fig. 2b). The rs373863828-A allele appears as one of the most extreme outliers on the chromosome, confirming that this locus exhibits an exceptionally strong signal of recent positive selection.

Next, we estimated the derived allele frequency trajectory with the CLUES program [44]. The allele frequency of rs373863828-A was found to have increased markedly over the past ~100 generations (Fig. 2c), with an estimated selection coefficient of 0.013. This result is consistent with our previous study of Oceanic populations, which suggested that rs373863828-A arose in the recent ancestors of Polynesians [18].

To assess whether similar patterns occurred at other previously reported BMI- or obesity-associated variants (Supplementary Table S1), we calculated iHS values and inferred allele frequency trajectories. In contrast to *CREBRF*, no evidence of recent positive selection was detected for the BMI-associated alleles rs34623097 of *ADRB2* and rs1137101 of *LEPR* (Supplementary Figs. S1, S2). Because the BMI-associated allele rs8192678-A of *PPRAGC1A* was not observed in the 22 Tongan individuals, this SNP was excluded from consideration in the present analysis and from all subsequent analyses.

BMI adaptation is thought to be influenced by multiple genetic factors [19–21]. To explore the potential impact of polygenic adaptation on the ‘thrifty’ genotype hypothesis, we reconstructed the historical trajectory of the population-average PS for BMI in Tongans using the GWAS summary data from East Asian populations [47].

Prior to interpreting historical polygenic score trajectories, we validated the applicability of East Asian GWAS data to our Tongan samples. Three linear regression models were fitted and compared: age only, PS only, and both age and PS as predictors of BMI. When focusing on a subset of 10 individuals younger than 45 years, the model incorporating both age and PS as explanatory variables provided a better fit than the model including age alone. In contrast, when analyzing all individuals, the model using age alone demonstrated superior performance (Supplementary Table S2). This finding is consistent with the notion that the predictive accuracy of polygenic scores tends to decline with increasing age [49, 50].

**Fig. 3:**
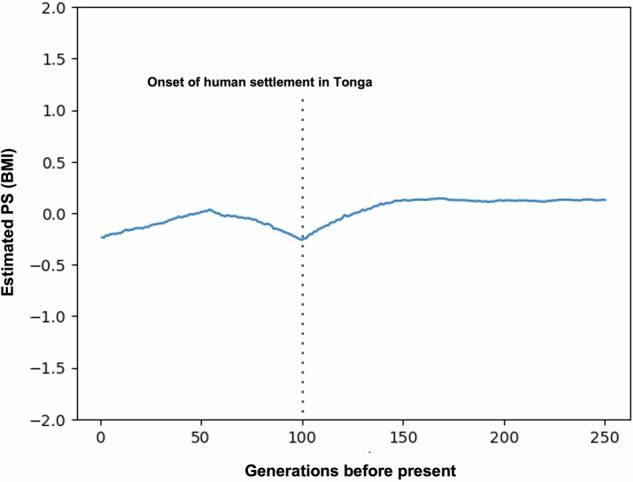
Polygenic Score (PS) trajectories in Tongan population. Time-series plot showing PS trajectories for BMI in the Tongan population, calculated using CLUES methodology across 250 generations before present. The dotted line shows the period of early human settlement in Tonga

Notably, the estimated population-average PS for BMI showed a gradual increase from 100 generations to 50 generations ago (Fig. 3), coinciding with the time period during which the ancestors of Polynesians migrated into Remote Oceania [6–8, 51]. In contrast, population-average PS began to decline from ~50 generations ago to the present (Fig. 3). To detect signals of recent polygenic selection on BMI and other BMI-related traits, we calculated tiHS, which represent iHS statistics weighted by the corresponding GWAS effect sizes [48]. Comparison of the tiHS distribution for BMI with the empirical null distribution revealed a strong signal of recent selection in the direction of decreasing BMI (Table 1 and Supplementary Fig. S3). When the same approach was applied to metabolism-related traits and blood biochemical parameters, several traits exhibited significant signals of decreasing selection (Table 1). Notably, type 2 diabetes (adjusted *P* = 5.3 × 10⁻³) and HDL cholesterol (adjusted *P* = 1.7 × 10⁻²) also showed evidence of selection acting in the decreasing direction, which is of particular interest given their metabolic relevance.

## Discussion

In this study, we aimed to examine whether natural selection has acted on obesity-related genetic variants in the ancestral population of Tongans and to evaluate potential genetic evidence supporting the thrifty gene hypothesis [1] in Remote Oceanic populations. The thrifty variant hypothesis proposes that genetic variants promoting efficient energy storage and fat accumulation conferred survival advantages during periods of food scarcity, particularly during long ocean voyages and colonization of remote islands. Based on this hypothesis, it is predicted that natural selection acted on such variants during the ancestral dispersal of Polynesians across the Pacific Ocean, leaving detectable signatures of selection and polygenic adaptation in the Tongan genome.

We demonstrated that positive selection acting on the rs373863828-A variant of *CREBRF* initiated its frequency increase in the ancestral population of Tongans ~100 generations ago (Fig. 2c). This variant, which is specific to Oceanian populations and associated with elevated BMI [17, 18], showed a clear signature of recent and strong selection in our Tongan samples. Notably, although signals of natural selection at this locus have been previously reported in Samoan populations [17], our findings extend this evidence to Tongans and provide temporal resolution by estimating the onset of selection at around 2800 years ago—coinciding with the period of early human settlement in Tonga [6–8, 51]. The presence of such a strong selection signal in both Samoans and Tongans suggests that rs373863828-A may have conferred adaptive advantages under shared ecological or demographic pressures during the peopling of Remote Oceania [17, 18]. Our results support the hypothesis that this allele was favored under conditions of food scarcity or intermittent availability, such as those encountered during long-distance voyaging or initial island colonization.

No clear signatures of natural selection were observed for the *ADRB2* and *LEPR* variants (Supplementary Fig. S1), which may primarily reflect their much older origins compared with *CREBRF* (Supplementary Fig. S4). These variants likely arose earlier in human evolution, and the haplotype-based signals of selection at these loci may have been progressively eroded by historical recombination over time.

The trajectory of population-average polygenic scores suggests that genome-wide genetic changes contributing to increased BMI occurred ~50–100 generations ago (1400–2800 years ago) (Fig. 3). This timeframe coincides with the proposed period of migration to Tonga [6–8, 51] and with the emergence of the positive selection acting on the *CREBRF* variant (Fig. 2c). These results indicate that, during this critical period, while positive selection was acting on the newly arisen *CREBRF* variant, allele frequency shifts in multiple pre-existing variants associated with BMI also occurred, possibly reflecting broader genetic changes influencing body composition.

Evidence of recent polygenic negative selection acting to reduce BMI was detected within the past 50 generations, as indicated by both the declining trajectory of population-average PS and the tiHS results (Table 1). This shift in selection pressure may reflect changing environmental conditions and lifestyle factors following European contact and subsequent modernization [52]. The high prevalence of obesity in the contemporary Tongan population may now represent a potential target of ongoing polygenic negative selection. Beyond BMI, natural selection also appears to have recently influenced other metabolic traits (Table 1), including reduced genetic susceptibility to type 2 diabetes, consistent with an adaptive shift toward improved metabolic efficiency. HDL cholesterol also showed evidence of decreasing selection, which may contribute to the elevated cardiovascular disease risk observed in present-day Tongans [25].

Several limitations of this study should be acknowledged. First, the PS for Tongans was calculated using GWAS data from East Asian populations, which may not adequately capture Oceania-specific variants [50, 53]. Second, the relatively small sample size (*n* = 22) limits the statistical power to detect subtle signals of selection and warrants cautious interpretation of the results. Future studies with expanded sample sizes will be essential to clarify the genetic architecture of BMI-related adaptations in Oceanic populations. Moreover, while our focus centered on BMI as a proxy for metabolic adaptation, alternative explanations for the observed selection patterns should be considered. Genetic variants that increased in frequency during ancestral migrations may have conferred selective advantages beyond energy storage, such as enhanced muscle mass development for physically demanding ocean voyages or improved thermoregulation during nighttime exposure at sea or moving between regions with different climates [54–56]. For instance, studies suggest that the rs373863828-A allele is associated with myostatin levels [57], and that its effect on BMI is primarily through lean mass rather than fat mass [58–61].

Our findings provide evidence that BMI-associated SNPs underwent positive selection in the ancestral Tongan population during their oceanic dispersal, which is likely considered an adaptive response to resource scarcity. The subsequent shift toward negative selection suggests that these once-beneficial adaptations may now contribute to increased susceptibility to obesity in modern environments. Together, these results offer evolutionary insights into how past selective pressures have shaped present-day genetic predispositions in Pacific Island populations.

## Supplementary information

Supplementary file
