# Supplementary material for: Polygenic and single-locus selection on BMI during Polynesian expansion
Source: J Hum Genet. 2025 Dec 15;71(5):293–300. doi: 10.1038/s10038-025-01441-y (PMC13109058; doi:10.1038/s10038-025-01441-y)
Supplement: Supplementary file 1 — Supplementary file [file 10038_2025_1441_MOESM1_ESM.docx]

**Supplementary figures and Tables**

# Polygenic and Single-Locus Selection on BMI During Polynesian Expansion

Hanako Miwa^1^, Mariko Isshiki^1,2^, Izumi Naka^1^, Ryosuke Kimura^3^, Tsukasa Inaoka^4^, Yasuhiro Matsumura^5^, Jun Ohashi^1,*^

^1^ Graduate School of Science, The University of Tokyo, Tokyo, Japan.

^2^ Department of Genetics, Albert Einstein College of Medicine, New York, United States of America.

^3^ Graduate School of Medicine, University of the Ryukyus, Okinawa, Japan.

^4^ Faculty of Agriculture, Saga University, Saga, Japan.

^5^ Faculty of Health and Nutrition, Bunkyo University, Kanagawa, Japan.

*Correspondence:

Jun Ohashi, Ph.D.

Graduate School of Science, The University of Tokyo

7-3-1 Hongo, Bunkyo-ku, Tokyo 113-0033, Japan

Email: jun_ohashi@bs.s.u-tokyo.ac.jp

**Supplementary figures**

**
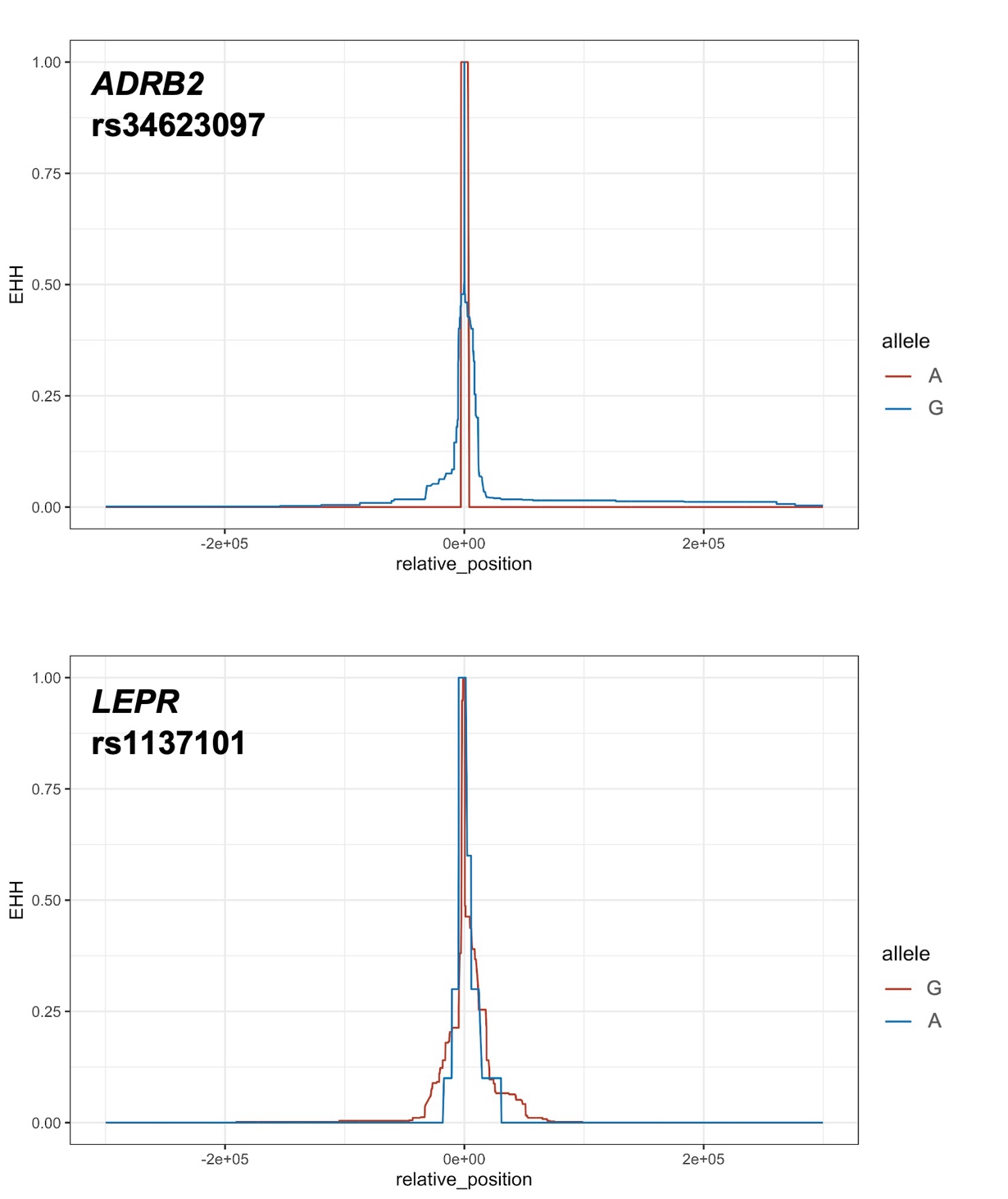
**

**Figure S1. Extended haplotype homozygosity plots for candidate genes.**

EHH decay plots are shown for four obesity-related candidate alleles: rs34623097-A (*ADRB2*): iHS = NA; rs1137101-A (*LEPR*): iHS = 0.67, p = 0.51. Red and blue lines represent the minor and major alleles at each SNP, respectively.


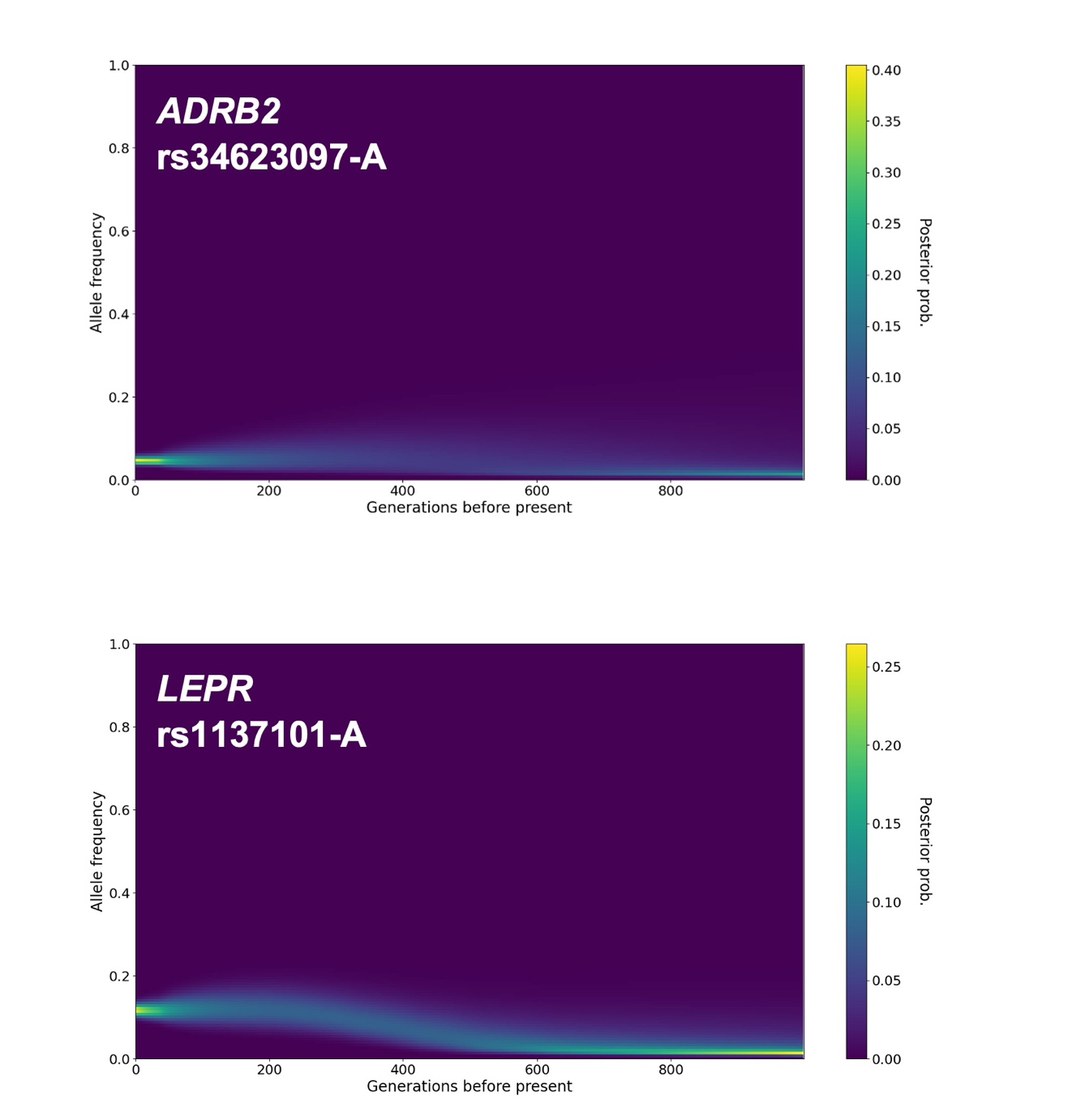


**Figure S2. Allele frequency trajectories for obesity-related genes.**

Heatmaps showing predicted allele frequency changes over 1,000 generations before present for four variants in candidate genes associated with obesity and metabolic traits: rs34623097-A (*ADRB2*); rs1137101-A (*LEPR*).


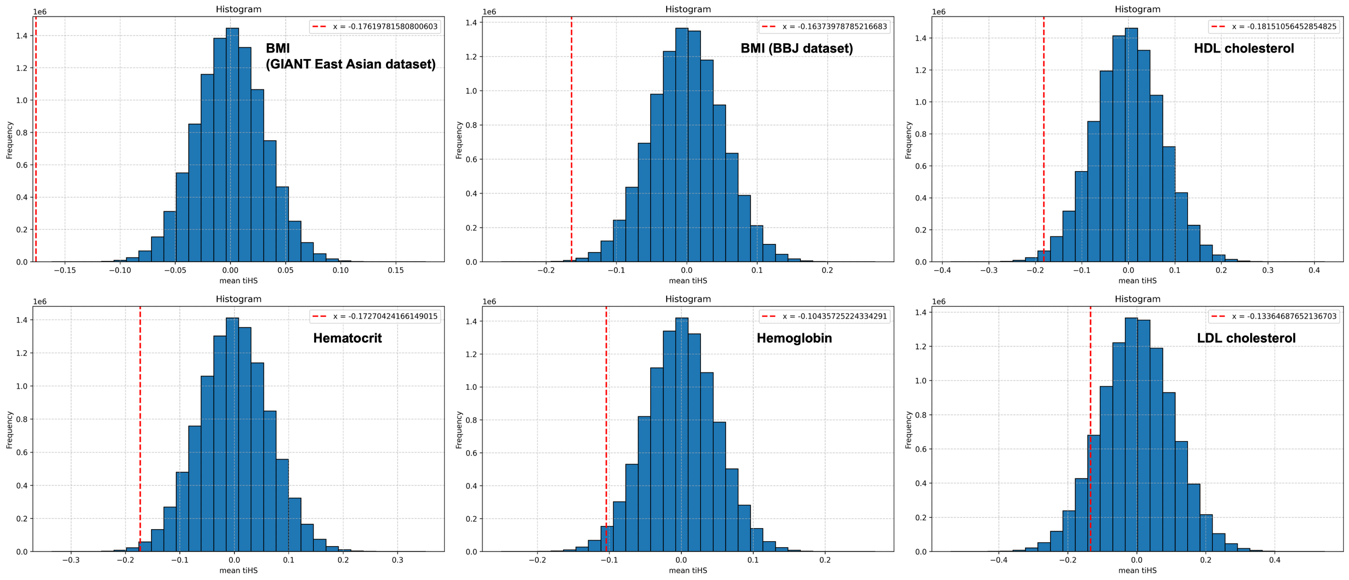


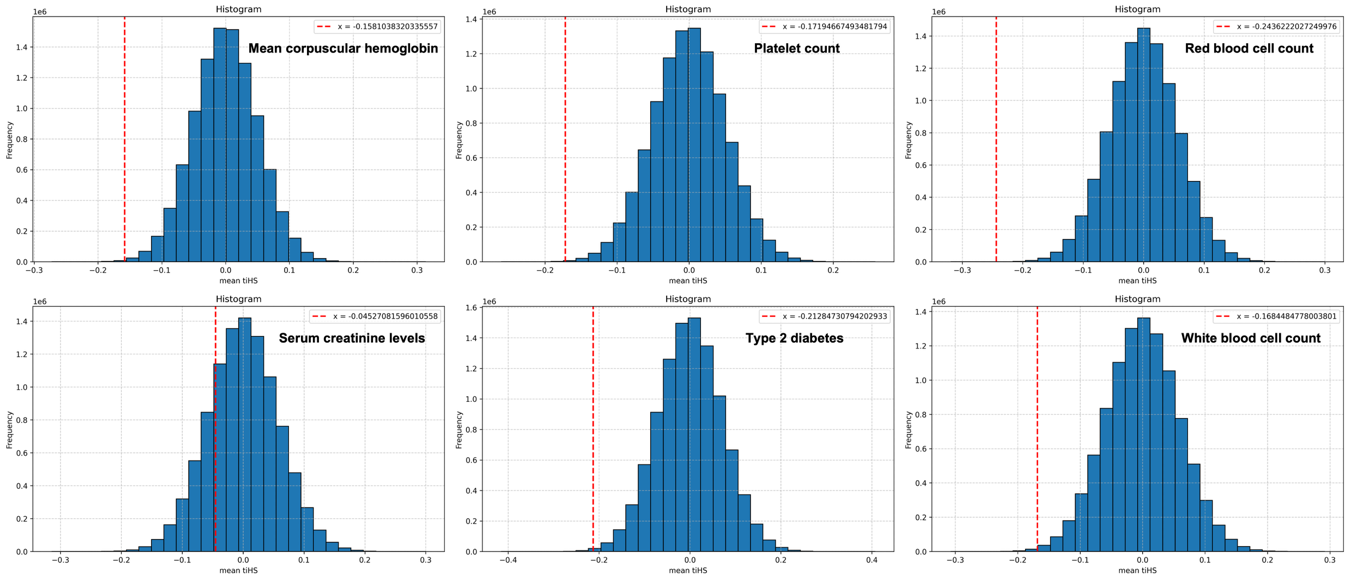


**Figure S3. Empirical distribution of mean tiHS values across randomly sampled SNP sets for metabolic and hematological traits.**

For each of the 12 metabolic and hematological traits, an empirical null distribution was generated by randomly sampling the same number of SNPs as in the trait-associated set, repeating this process 10,000,000 times. The resulting histograms illustrate these null distributions. The red dotted line indicates the observed mean tiHS value calculated using the GWAS effect sizes (Table 1). HDL, high-density lipoprotein; LDL, low-density lipoprotein; BMI, body mass index.


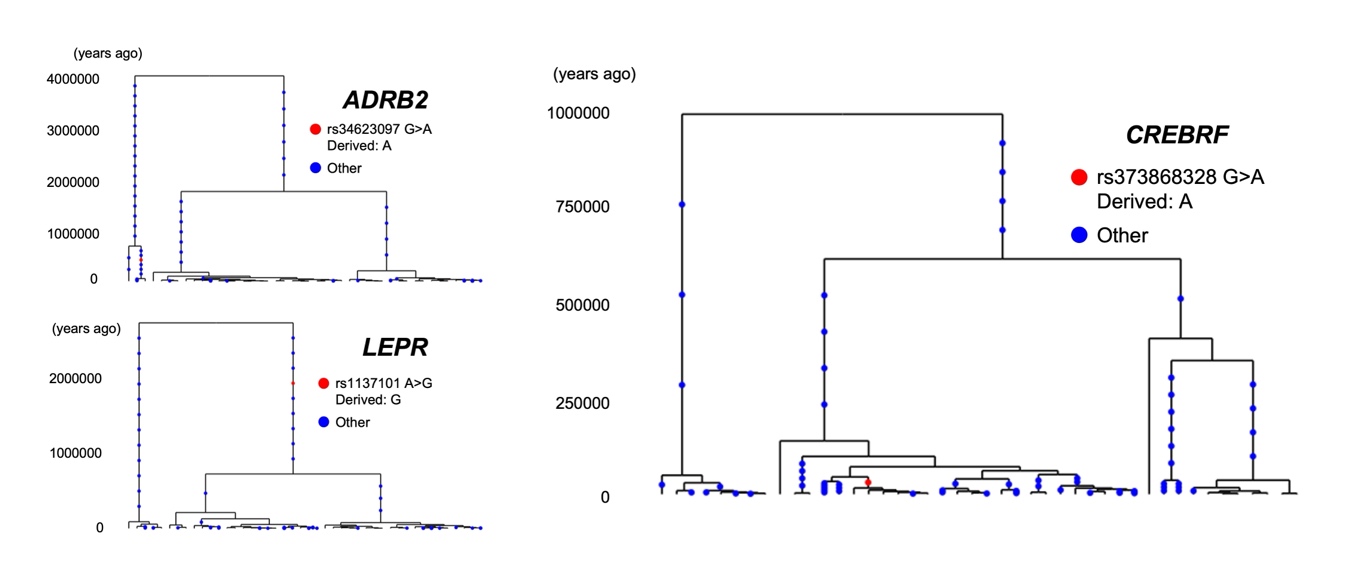


**Figure S4. Genealogical trees for candidate obesity-related loci.**

Genealogical trees inferred using Relate software for the five candidate variants: rs373863828-A (*CREBRF*); rs34623097-A (*ADRB2*); rs1137101-G (*LEPR*). Trees were constructed assuming 28 years per generation, and the time scale is shown in years.

**Supplementary tables**

**Table S1. BMI-associated variants studied in this study.**

| **SNP** | **Gene** | **Ancestral allele** | **Derived allele** | **BMI-associated allele** | **Frequency (Tonga)** | **Frequency (East Asia)** |
| --- | --- | --- | --- | --- | --- | --- |
| rs373863828 | *CREBRF* | G | A | A | 0.14 | 0.00 |
| rs34623097 | *ADRB2* | G | A, C | A | 0.05 | 0.07 |
| rs1137101 | *LEPR* | A | G, T | A | 0.11 | 0.13 |
| rs8192678 | *PPARGC1A* | T | C, A, G | A | 0.00 | 0.00 |

**Note:** Frequencies for “Tonga” and “East Asia” indicate the allele frequencies of the BMI-associated allele. “East Asia” represents the combined data from CHB, JPT, CHS, CDX, and KHV populations in the 1000 Genomes Project.

**Table S2. Linear regression models for BMI prediction.**

| **Models (age group*)** | **AIC** | **BIC** | **adj R^2^** | **coef (AGE)** | **coef (PS)** |
| --- | --- | --- | --- | --- | --- |
| BMI ~ AGE (all) | 142.9 | 145.1 | 0.349 | 0.331 | - |
| BMI ~ PS (all) | 153.4 | 155.6 | -0.047 | - | -0.229 |
| BMI ~ PS + AGE (all) | 144.8 | 148.1 | 0.319 | 0.332 | -0.274 |
| BMI ~ AGE (< 55) | 89.62 | 91.04 | 0.501 | 0.401 | - |
| BMI ~ PS (< 55) | 101.1 | 102.5 | -0.074 | - | -0.364 |
| BMI ~ PS + AGE (< 55) | 90.94 | 93.07 | 0.484 | 0.411 | -0.944 |
| BMI ~ AGE (< 50) | 67.87 | 68.84 | 0.650 | 0.505 | - |
| BMI ~ PS (< 50) | 81.05 | 82.02 | -0.050 | - | 1.426 |
| BMI ~ PS + AGE (< 50) | 68.11 | 69.56 | 0.664 | 0.575 | -1.597 |
| BMI ~ AGE (< 45) | 42.76 | 43.37 | 0.878 | 0.538 | - |
| BMI ~ PS (< 45) | 61.00 | 61.61 | 0.243 | - | 4.411 |
| BMI ~ PS + AGE (< 45) | 35.81 | 36.71 | 0.943 | 0.483 | 2.091 |

*Each model was fitted to a different age-based subset of the 22 Tongan individuals. Sample sizes: all (n = 22), <55 (n = 15), <50 (n = 12), <45 (n = 10).
